# International Medical Congress

**Published:** 1890-05

**Authors:** 


### International Medical Congress.

The Committee of Organization of the Tenth International Medical Congress are, Vierchaw, President; E. Von Bergman, E. Leyden, W. Waldeyer, Vice-Presidents; O. Lasson, Secretary General, with sub-committees, are energetically working in perfecting arrangements for the congress. They earnestly desire the sympathy and co-operation of the profession in America.

The delegates of American medical societies and institutions, and individual members of the profession, will be admitted on equal terms, and it is to be hoped that a large number of distinguished men of our country will appreciate the honor conferred by this cordial invitation, and the opportunity afforded to fitly represent American medicine. The congress promises to be of very great value in its educational results, and in securing the ties of international brotherhood. It is important that the American profession should participate both in its labors and its fruits. The congress is to be held at Berlin, from the 4th to the 9th of August.

The arrangements in regard to a full general meeting and the main scientific work which is delegated to the sections are the same as in former sessions.

A medico-scientific exhibition, the programme of which has been published, is to form an ingredient part; it is the latter that the Berlin Committee is very anxious that both the scientific and secular press should be requested to give the greatest publicity. The office of the secretary general is in Karl Strasse, No. 19 W. Berlin. It will be remembered that the dental section is on exactly the same footing as that of other sections of the congress. This general invitation is extended to dentists exactly as to those of other specialties, or to the general practitioner.

We hope to see a large representation of the members of our profession at the meeting of the congress. We would suggest that each one, as soon as he decides to go, would remit his initiation fee, $5, to the secretary general. This would avoid delay and difficulty in registration after arriving there. The history of the past two congresses in this matter of registration emphasizes this suggestion, especially to all those familiar with the circumstances of those occasions.

Exchanges, Books, Etc.—From the fact that some of our exchanges and books sent for notice are long delayed in their coming, and sometimes do not reach us at all, we take this method of earnestly requesting that all exchanges, books, etc., intended for the Editor of the Register be sent directly to his address, No. 122 W. Seventh street, Cincinnati. We hope this request will receive the attention of all interested.

Quizs—Compends.—We have just received a list of eleven volumes of Quiz Compends, new editions, and just issued by P. Blakistone, Son & Co., of Philadelphia. These are for the use of students and physicians; they are based upon the most popular text books, and the lectures of prominent professors.

These volumes have been prepared and thoroughly revised by quiz masters and attaches of colleges who are well acquainted with the wants of students. They are of such a character as to be valuable to the students of all medical and dental colleges. Interleaved copies may be obtained by taking notes. They are for sale by book sellers generally.

Correction.—In the April number of the Register, page 192, in the fifteenth line from the bottom of page, for the word *ivory*, read *work*.

